# Hepatocyte-Specific Depletion of UBXD8 Induces Periportal Steatosis in Mice Fed a High-Fat Diet

**DOI:** 10.1371/journal.pone.0127114

**Published:** 2015-05-13

**Authors:** Norihiro Imai, Michitaka Suzuki, Kazuhiko Hayashi, Masatoshi Ishigami, Yoshiki Hirooka, Takaya Abe, Go Shioi, Hidemi Goto, Toyoshi Fujimoto

**Affiliations:** 1 Department of Anatomy and Molecular Cell Biology, Nagoya University Graduate School of Medicine, Nagoya, Japan; 2 Department of Gastroenterology and Hepatology, Nagoya University Graduate School of Medicine, Nagoya, Japan; 3 Laboratory for Animal Resources and Genetic Engineering, RIKEN Center for Developmental Biology, Kobe, Japan; University of Basque Country, SPAIN

## Abstract

We showed previously that UBXD8 plays a key role in proteasomal degradation of lipidated ApoB in hepatocarcinoma cell lines. In the present study, we aimed to investigate the functions of UBXD8 in liver *in vivo*. For this purpose, hepatocyte-specific UBXD8 knockout (UBXD8-LKO) mice were generated. They were fed with a normal or high-fat diet, and the phenotypes were compared with those of littermate control mice. Hepatocytes obtained from UBXD8-LKO and control mice were analyzed in culture. After 26 wk of a high-fat diet, UBXD8-LKO mice exhibited macrovesicular steatosis in the periportal area and microvesicular steatosis in the perivenular area, whereas control mice exhibited steatosis only in the perivenular area. Furthermore, UBXD8-LKO mice on a high-fat diet had significantly lower concentrations of serum triglyceride and VLDL than control mice. A Triton WR-1339 injection study revealed that VLDL secretion from hepatocytes was reduced in UBXD8-LKO mice. The decrease of ApoB secretion upon UBXD8 depletion was recapitulated in cultured primary hepatocytes. Accumulation of lipidated ApoB in lipid droplets was observed only in UBXD8-null hepatocytes. The results showed that depletion of UBXD8 in hepatocytes suppresses VLDL secretion, and could lead to periportal steatosis when mice are fed a high-fat diet. This is the first demonstration that an abnormality in the intracellular ApoB degradation mechanism can cause steatosis, and provides a useful model for periportal steatosis, which occurs in several human diseases.

## Introduction

Steatosis (fatty liver) is highly prevalent in Western countries and may lead to steatohepatitis, cirrhosis, and liver cancer [1,2]. Western-style diets, as well as metabolic disorders such as obesity, insulin resistance, and type 2 diabetes mellitus, have been characterized as major risk factors [3,4], but other abnormalities can also cause steatosis, either on their own or in combination with other conditions [1]. Genetically manipulated mouse models have shown that defects in lipid metabolism are conductive to steatosis and/or steatohepatitis [5].

Hepatocytes in steatotic liver harbor a large number of lipid droplets (LDs) in the cytoplasm. The LD is an organelle that exists in many types of cells and stores lipid esters, mainly triglycerides (TG) and cholesterol esters, which are mobilized for various physiological processes such as β-oxidation and membrane biogenesis [6,7]. Thus, the presence of LDs is important for normal cell physiology. However, the excess LDs in steatotic liver indicate some abnormality that could be related to overproduction of lipid esters or a deficiency in lipid ester hydrolysis and use.

In hepatocytes, lipid esters stored in LDs are utilized for synthesis of very low-density lipoproteins (VLDL) [8]. VLDL formation occurs by co-translational and post-translational lipidation of apolipoprotein B (ApoB), and mature VLDL is secreted as a lipid ester–laden particle containing an ApoB molecule [9]. For synthesis of VLDL, fatty acids (FAs) generated by hydrolysis of lipid esters in LDs are used preferentially over FAs that are newly synthesized or incorporated from the extracellular milieu [8]. Due to this preference for lipids stored in LDs, a decrease in VLDL synthesis and/or secretion may lead to excessive accumulation of LDs in hepatocytes. For example, patients with hypobetalipoproteinemia, who have low plasma VLDL levels due to genetic abnormalities in the ApoB molecule, are susceptible to steatosis [10].

Because of its importance in systemic lipid homeostasis, VLDL secretion from hepatocytes is tightly regulated, primarily through degradation of ApoB proteins rather than transcriptional regulation. Proteasomal degradation of poorly lipidated ApoB at the Sec61 translocon has been characterized in detail, but ApoB after lipidation is also degraded in the ER or post-ER compartments [11,12,13]. We previously showed that lipidated ApoB is subjected to ubiquitination and degradation in the vicinity of LDs, and that UBXD8 plays a major role in this process [14,15,16]. UBXD8, a hairpin-shaped protein anchored to the cytoplasmic surface of LDs and the ER, binds to p97/VCP and ubiquitinated proteins through its UBX and UBA domains, respectively [16,17]. In the hepatocarcinoma cell line Huh7, UBXD8 is engaged in transporting ubiquitinated ApoB to proteasomes, and knockdown of UBXD8 causes aberrant accumulation of ApoB in the ER lumen facing LDs [16].

Our previous result led us to hypothesize that dysfunction of UBXD8 may cause abnormalities in systemic lipid metabolism. In this study, in order to address that possibility in the physiological setting, we generated a hepatocyte-specific UBXD8-deficient mouse and studied its phenotype in comparison to normal littermates. UBXD8-deficient mice fed a high-fat diet developed macrovesicular steatosis in the periportal area (zone 1) of the liver lobule, whereas control mice did not. Moreover, hepatocyte-specific UBXD8-deficient mice had significantly lower levels of serum VLDL and TG than control mice. These results indicated that deficiency of UBXD8 in hepatocytes compromises LD functionality, decreases VLDL secretion, and predisposes to steatosis.

## Materials and Methods

### Antibodies

Rabbit anti-UBXD8 antibody (GeneTex, Irvine, CA), goat anti-albumin antibody (Bethyl Laboratories, Montgomery, TX), goat anti–apolipoprotein B antibody (Rockland Immunochemicals, Limerick, PA), rabbit anti–β-actin antibody (Sigma–Aldrich, St. Louis, MO), and secondary antibodies conjugated to horseradish peroxidase (Thermo Fisher Scientific, Waltham, MA) or fluorochromes (Thermo Fisher Scientific; Jackson ImmunoResearch Lab, West Grove, PA) were obtained from the indicated suppliers.

### Animals

**Fig 1:**
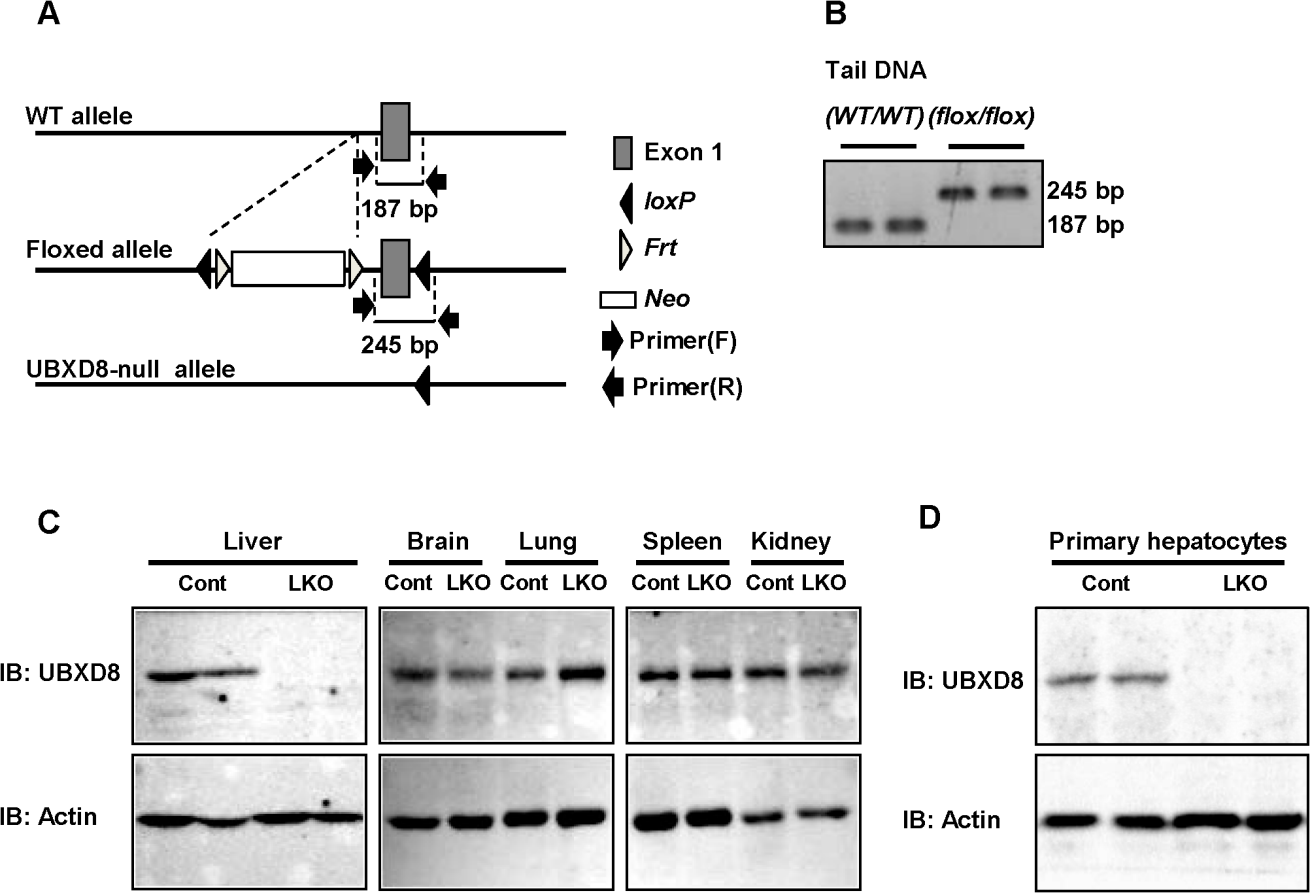
Hepatocyte-specific deletion of UBXD8 in mice. (A) PCR genotyping. The 187-bp and 245-bp products were obtained from wild-type and *Ubxd8*flox/flox mice, respectively. (B) Western blot showing liver-specific depletion of UBXD8 in the UBXD8-LKO mouse. Tissue lysates were prepared from control (*Cre*-/-, *Ubxd8*flox/flox) and UBXD8-LKO (*Cre*+/-, *Ubxd8*flox/flox) mice, and an equal amount (30 μg) of protein was loaded for each lane in SDS-PAGE. (C) Western blot showing depletion of UBXD8 in hepatocytes isolated from the UBXD8-LKO mouse.

Mice with exon 1 of *Ubxd8* flanked by two *LoxP* sites (*Ubxd8* ^*flox/flox*^) (Accession No. CDB0956K: http://www.cdb.riken.jp/arg/mutant%20mice%20list.html) (A) were generated in a mixed genetic background of C57BL/6N and CBA, as described (http://www.cdb.riken.jp/arg/Methods.html). The *Ubxd8* ^*flox/flox*^ mice were crossed with mice expressing the Cre recombinase driven by the albumin promoter (Jackson Laboratory, Bar Harbor, ME; B6.Cg-Tg(Alb-cre)21Mgn/J) to generate hepatocyte-specific UBXD8 knockout (UBXD8-LKO) mice (Fig 1A). The UBXD8-LKO mice and their age-matched littermate controls (*Cre* ^*-/-*^, *Ubxd8* ^*flox/flox*^) were maintained in temperature- and humidity-controlled specific pathogen-free conditions with a 12-h light/dark cycle and free access to food and water.

Both UBXD8-LKO and the control mice were divided into two dietary groups: (1) normal chow diet (ND: CLEA Rodent Diet CE-2, 4.6% fat, CLEA Japan, Tokyo, Japan), and (2) high-fat diet (HFD: CLEA Rodent Diet Quick Fat, 14.4% fat, CLEA Japan). The diets started when the mice were 4 wk of age and continued until the end of the experiments. The mice were anesthetized by intraperitoneal injection of sodium pentobarbital (50 mg/kg; Somnopentyl, Kyoritsu Seiyaku, Tokyo, Japan). All animal experiments conformed to the Guidelines for Proper Conduct of Animal Experiments of the Science Council of Japan and were approved by the Animal Experimentation Committee of the Nagoya University Graduate School of Medicine (Approval ID: 26211).

### PCR

Genotyping of *Ubxd8* ^*flox/flox*^ mice was performed by PCR of tail genomic DNA obtained from 4-wk-old mice (Fig 1A and 1B). Primers were as follows: for loxP, forward primer: 5’-CCAACCTCTGTGGGTCCTC-3’; reverse primer: 5’-TAACTCAGATCCCCAATCGC-3’; for albumin-Cre, forward primer: 5’-TTTGCCTGCATTACCGGTCGATGCAAC -3’; reverse primer: 5’-TGCCCCTGTTTCACTATCCAGGTTACGGA-3’. Because tail genomic DNA was identical between the control (*Cre* ^*-/-*^, *Ubxd8* ^*flox/flox*^) and UBXD8-LKO (*Cre* ^*+/-*^, *Ubxd8* ^*flox/flox*^) mice, verification of UBXD8 knockout in liver was performed by Western blotting, as described below.

### Morphological analysis of liver

A small piece of liver from the left lateral lobe was excised from anesthetized mice after a 12-h fast and exsanguination; the samples were fixed in 10% neutral buffered formalin. Paraffin sections were stained with hematoxylin and eosin (H&E) or Sirius red and observed using a BZ-X700 microscope (Keyence, Osaka, Japan). Micrographs were collected from randomly chosen 1 mm × 1.5 mm areas. Samples were classified as exhibiting perivenular microvesicular steatosis when more than 10% of hepatocytes in zones 2 and 3 harbored small vesicles, and as exhibiting periportal macrovesicular steatosis when more than 30% of periportal zones (zone 1) contained hepatocytes harboring large vesicles.

For electron microscopy, liver was perfusion-fixed with 2.5% glutaraldehyde and 2% formaldehyde in 0.1 mol/l sodium cacodylate buffer, post-fixed with 1% osmium tetroxide and 0.1% potassium ferrocyanide in the same buffer, dehydrated, and embedded in Quetol-812. Ultrathin sections counterstained with uranyl acetate and lead citrate were observed using a JEOL1011 electron microscope (JEOL, Tokyo, Japan).

### Biochemical analysis of blood samples

Blood samples were collected from 30-wk-old mice after 12 h of fasting. Serum levels of total protein, albumin, total bilirubin, glucose, TG, phospholipid, free FAs, total cholesterol, esterified cholesterol, free cholesterol, aspartate aminotransferase (AST), alanine aminotransferase (ALT), lactate dehydrogenase, and alkaline phosphatase (ALP) were measured by SRL (Tokyo, Japan). Analysis of serum lipoprotein profiles using gel-filtration high-performance liquid chromatography (HPLC; LipoSEARCH) was performed by Skylight Biotech (Akita, Japan) [18]. The size of lipoproteins was defined as follows: CM, > 80 nm; VLDL, 30–80 nm; LDL, 16–30 nm; HDL, 8–16 nm [19].

### Analysis of liver lipids

Lipids were extracted from frozen liver specimens with a mixture of chloroform/methanol (2:1) using Folch’s method [20]. Concentrations of TG, total cholesterol, and free cholesterol were assayed enzymatically using the TG-E test colorimetric kit, T-Cho E kit (Wako Pure Chemical Industries, Osaka, Japan), and Determiner L FC kit (Kyowa Medex, Tokyo, Japan), respectively.

### VLDL secretion assay

To estimate lipoprotein secretion from liver, mice fasted for 2 h were injected with Triton WR-1339 (500 mg/kg body weight; Sigma–Aldrich) to inhibit lipoprotein lipase [21]. Blood samples were obtained from the tail vein before and 1 and 2 h after the Triton WR-1339 injection. Serum TG was measured by enzymatic methods using the TG-E test colorimetric kit.

### Western blotting

Proteins extracted from mouse tissue were electrophoresed by SDS-PAGE and electrotransferred to nitrocellulose filters. The Western blot signal was converted to chemiluminescence and captured on a Light-Capture II imager (ATTO, Tokyo, Japan). The relative intensity of signals was measured using CS Analyzer 3 software (ATTO).

### Cells and transfection

HepG2 cells were obtained from the Japanese Collection of Research Bioresources Cell Bank and cultured in Dulbecco’s modified Eagle’s medium (DMEM) supplemented with 10% fetal bovine serum (FBS) and antibiotics at 37°C in a humidified atmosphere containing 5% CO_2_. Oleic acid (OA; Sigma-Aldrich) was mixed with FA-free bovine serum albumin (BSA) (Wako Pure Chemical Industries, Osaka, Japan) at a molar ratio of 6:1 and applied at a final concentration of 0.4 mmol/l. Cells were transfected with small interfering RNA (siRNA) with Lipofectamine 2000 (Thermo Fisher Scientific).

### Primary hepatocytes in culture

Hepatocytes were isolated from mouse liver by the two-step collagenase perfusion method [22,23]. Briefly, Hank’s buffered salt solution followed by collagenase digestion medium was perfused through a cannula inserted from superior vena cava. After the viability was checked, 3 × 10^6^ cells were plated in a 60-mm dish in DMEM supplemented with 10% FBS and antibiotics, and kept under an atmosphere of 95% air and 5% CO_2_ at 37°C.

ApoB secretion was analyzed using cells 1 day after the isolation. The culture medium was replaced with fresh medium, and then collected at 0, 1, 3, 6, 12, and 24 h. Samples were subjected to SDS-PAGE, western blotting, and densitometry to quantitate the amount of ApoB secreted into the medium. ApoB levels were normalized against the number of cells each sample, calculated from the amount of albumin secreted into the culture medium.

For immunofluorescence labeling of ApoB, cells were fixed with a mixture of 3% formaldehyde and 0.01% glutaraldehyde in 0.1 mol/l phosphate buffer for 15 min, and then permeabilized with 0.01% digitonin in PBS for 30 min before blocking and incubation with goat anti-ApoB antibody (Bethyl Laboratories). LDs and nuclei were stained with BODIPY493/503 (Thermo Fisher Scientific) and bisBenzimide H33342 trihydrochloride (Hoechist, Sigma-Aldrich), respectively. Images were captured using an Axiovert 200M fluorescence microscope (Carl Zeiss, Jena, Germany) equipped with an Apochromat 63X lens with a 1.40 numerical aperture. Images were obtained using the Apotome processing system.

### Quantitative real time-PCR analysis

Total RNA was isolated from frozen liver with TRIzol reagent (Thermo Fisher Scientific) and reverse transcribed into cDNA using ReverTraAce qPCR kit (TOYOBO, Osaka, Japan). Expression analysis was performed by quantitative real-time PCR with Brilliant 3 Ultra-Fast SYBR Green QPCR master mix (Agilent Technologies, Santa Clara, CA), using *Hprt* as the internal control. The TaqMan probes used for PCR amplification were obtained from Applied Biosystems (Thermo Fisher Scientific).

### DNA microarray analysis

DNA microarray analysis was performed by Kurabo Industries. Briefly, total RNA cleaned up with the RNeasy MinElute Cleanup Kit (QIAGEN, Hilden, Germany) was applied to GeneChip Mouse Gene ST Array (Affymetrix, Santa Clara, CA). Scanning was performed on a GeneChip Scanner 3000 (Affymetrix), and the image was analyzed using GeneChip Operating Software (ver1.4) and Expression Console Software ver.1.2.1 (Affymetrix).

### Pulse-chase experiment

HepG2 cells transfected with control or UBXD8 siRNA (GE Healthcare Bio-Sciences, Piscataway, NJ) were incubated in methionine/cysteine-free DMEM for 60 min, pulsed with 35 mCi/ml ^35^S-methionine/cysteine (PerkinElmer, Waltham, MA) for 30 min, and chased with cold methionine/cysteine for various periods of time. OA (0.4 mmol/l) in complex with FA-free BSA was administered simultaneously with the pulse label and kept in the culture medium during the chase period. ApoB immunoprecipitated from the medium and cell lysate was subjected to Western blotting and quantitated using a Typhoon scanner (GE Healthcare Bio-Sciences).

### Statistical analysis

Statistical significance was analyzed either by Fisher’s exact test or Student’s *t* test, as appropriate, using SPSS ver. 20 (IBM, Armonk, NY). All data are expressed as means ± SEM. All tests were two-sided, and P values less than 0.05 were considered to represent statistically significant differences.

### Box plots

Box plots were prepared using BoxPlotR, provided at http://boxplot.tyerslab.com/ [24].

## Results

### Generation of hepatocyte-specific UBXD8 knockout mouse

UBXD8-LKO mice (*Cre* ^*+/-*^, *Ubxd8* ^*flox/flox*^) were born at Mendelian ratios and grew without exhibiting any outward differences from littermate controls (*Cre* ^*-/-*^, *Ubxd8* ^*flox/flox*^). Over the course of repeated matings between UBXD8-LKO and control mice, the conditional knockout animals were born at the same rate as controls [UBXD8-LKO:control = 120:141 (female), 122:140 (male)], demonstrating that liver-specific deletion of UBXD8 did not significantly influence general development. Western blotting analysis confirmed lack of UBXD8 expression in the liver of UBXD8-LKO mice, whereas in other organs, including brain, lung, spleen, and kidney, UBXD8 was expressed at levels identical to those in controls (Fig 1C). The absence of UBXD8 expression in hepatocytes per se was confirmed by using primary hepatocytes obtained from UBXD8-LKO mice (Fig 1D). The result showed that UBXD8-LKO mice lacked UBXD8 expression specifically in hepatocytes.

### UBXD8-LKO mice fed a normal diet did not differ significantly from controls

**Fig 2:**
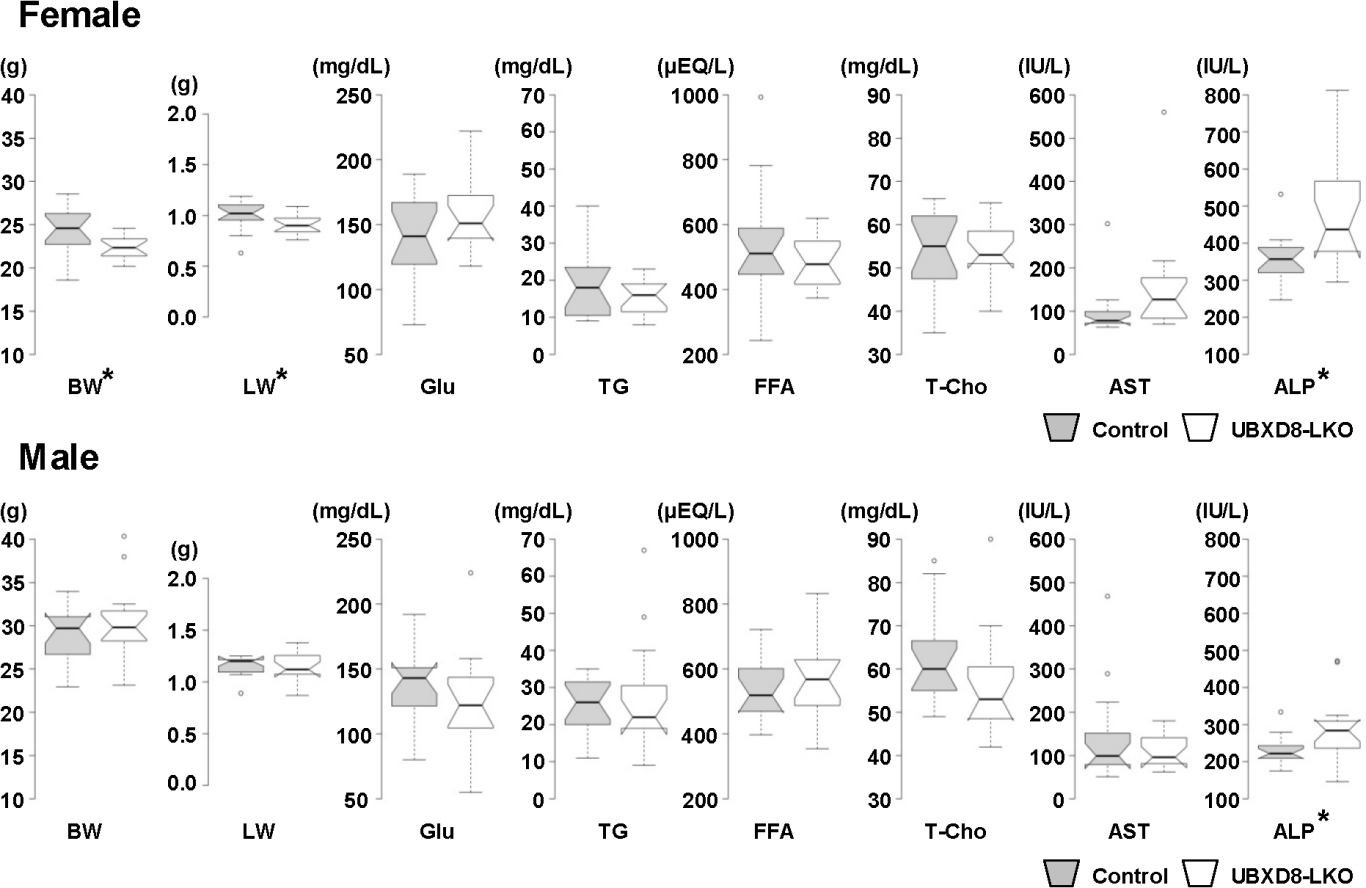
Body weight, liver weight, and blood chemistry results of mice fed a normal diet. Control and UBXD8-LKO mice (30 wk old) were fed a normal diet for 26 wk. 15 females (A) and 15 males (B) were examined for each group. Center lines show the medians; box limits indicate the 25th and 75th percentiles; whiskers extend 1.5 times the interquartile range (IQR) from the 25th and 75th percentiles; and outliers are represented by dots. The notches are defined as ± 1.58*IQR/sqrt(n) and represent the 95% confidence interval for each median. Indices marked with an asterisk (*) differed significantly between the two groups (P < 0.05; non-paired Student's t test). Numerical data (means ± SEM) and data on additional indices are shown in Table 1. BW: body weight; LW: liver weight; Glu: glucose; FFA: free fatty acid; T-Cho: total cholesterol.

UBXD8-LKO mice and littermate controls were kept on a normal diet for 26 wk after weaning, and 15 males and 15 females from each group (i.e., 60 mice in total) were examined at 30 wk of age. As shown in Fig 2 and Table 1, UBXD8-LKO and control mice exhibited statistically significant differences in body weight, liver weight, and serum total protein in females and serum phospholipid in males. For all four indices, however, the difference was relatively small, and a difference in the opposite direction was seen in mice of the other sex. For example, serum phospholipid level in males was lower in UBXD8-LKO mice than in controls, whereas in females it was higher in UBXD8-LKO.

**Table 1:** Body weight, liver weight, and blood chemistry results of mice fed a normal diet.

Normal diet						
	Female			Male		
	Control	UBXD8-LKO	P value	Control	UBXD8-LKO	P value
**Parameter**						
**Body weight (g)**	**24.16±0.69**	**22.33±0.36**	0.026*	**29.08±0.80**	**30.54±1.10**	**0.292**
**Liver weight (g)**	**1.01±0.03**	**0.90±0.02**	0.030*	**1.14±0.02**	**1.15±0.03**	**0.77**
**Liver/Body ratio (%)**	**4.17±0.08**	**4.06±0.07**	**0.333**	**3.94±0.10**	**3.81±0.09**	**0.349**
**Serum parameter**						
**Total protein (g/dL)**	**4.86±0.09**	**4.56±0.06**	0.012*	**4.68±0.05**	**4.54±0.05**	**0.064**
**Albumin (g/dL)**	**3.27±0.04**	**3.20±0.03**	**0.251**	**3.03±0.04**	**2.97±0.04**	**0.368**
**Total bilirubin (mg/dL)**	**0.072±0.008**	**0.094±0.010**	**0.101**	**0.070±0.004**	**0.068±0.004**	**0.749**
**Glucose (mg/dL)**	**142.00±8.79**	**157.93±7.65**	**0.183**	**135.07±7.36**	**126.20±9.93**	**0.479**
**Triglyceride (mg/dL)**	**19.20±2.51**	**15.87±1.19**	**0.242**	**24.60±2.15**	**26.67±3.94**	**0.649**
**Phospholipid (mg/dL)**	**109.93±4.28**	**117.67±3.78**	**0.187**	**138.67±4.64**	**123.20±5.61**	0.043*
**Free fatty acid (μEQ/L)**	**536.93±46.19**	**482.27±20.01**	**0.287**	**531.13±23.81**	**577.67±34.21**	**0.274**
**Total cholesterol (mg/dL)**	**53.53±2.59**	**54.00±1.85**	**0.885**	**62.93±2.88**	**55.87±3.14**	**0.109**
**Esterified cholesterol (mg/dL)**	**39.67±2.10**	**38.87±1.42**	**0.755**	**46.87±2.19**	**41.47±2.24**	**0.097**
**Free cholesterol (mg/dL)**	**13.87±0.63**	**15.13±0.55**	**0.144**	**16.07±0.88**	**14.40±0.97**	**0.216**
**Aspartate aminotransferase (IU/L)**	**98.07±15.29**	**156.07±31.53**	**0.109**	**143.80±28.36**	**109.07±10.31**	**0.26**
**Alanine aminotransferase (IU/L)**	**30.53±2.33**	**54.27±12.94**	**0.082**	**37.47±3.77**	**37.60±2.55**	**0.977**
**Lactate Dehydrogenase (IU/L)**	**439.93±79.38**	**384.60±45.71**	**0.551**	**573.47±77.05**	**499.47±34.49**	**0.388**
**Alkaline phosphatase (IU/L)**	**358.53±17.10**	**484.73±37.08**	0.004*	**229.73±10.09**	**291.27±22.07**	0.017*

Comparison of control and UBXD8-LKO mice (30 wk old) fed a normal diet. For each group, 15 females and 15 males were examined. P values were obtained by non-paired Student's t test. Selected data sets are presented as box plots in Fig 2. n = 15. All data are presented as mean ± SE.

P values are the results of non-paired Student's t test

(*: P<0.05).

On the other hand, the serum ALP level in the UBXD8-LKO mouse was 1.35-fold higher in females and 1.27-fold higher in males. This observation might suggest minor damage in UBXD8-null hepatocytes; however, the levels of other enzymes that should also increase in serum upon liver damage, i.e., AST, ALT, and lactate dehydrogenase, did not change. Therefore, it seemed unlikely that liver damage had occurred. Moreover, histological analysis of liver paraffin sections stained with H&E did not reveal any difference between UBXD8-LKO and control mice or any sign of hepatic damage or steatosis (S1 Fig). This result confirmed that depletion of UBXD8 alone did not cause significant changes in liver histology and function up to 30 wk of age when mice were fed a normal diet. We maintained several mice from both cohorts to 65 wk of age on the normal diet, but they did not exhibit particular histological differences or abnormalities in their blood tests (data not shown).

### UBXD8-LKO mice fed a high-fat diet developed periportal macroscopic steatosis

**Fig 3:**
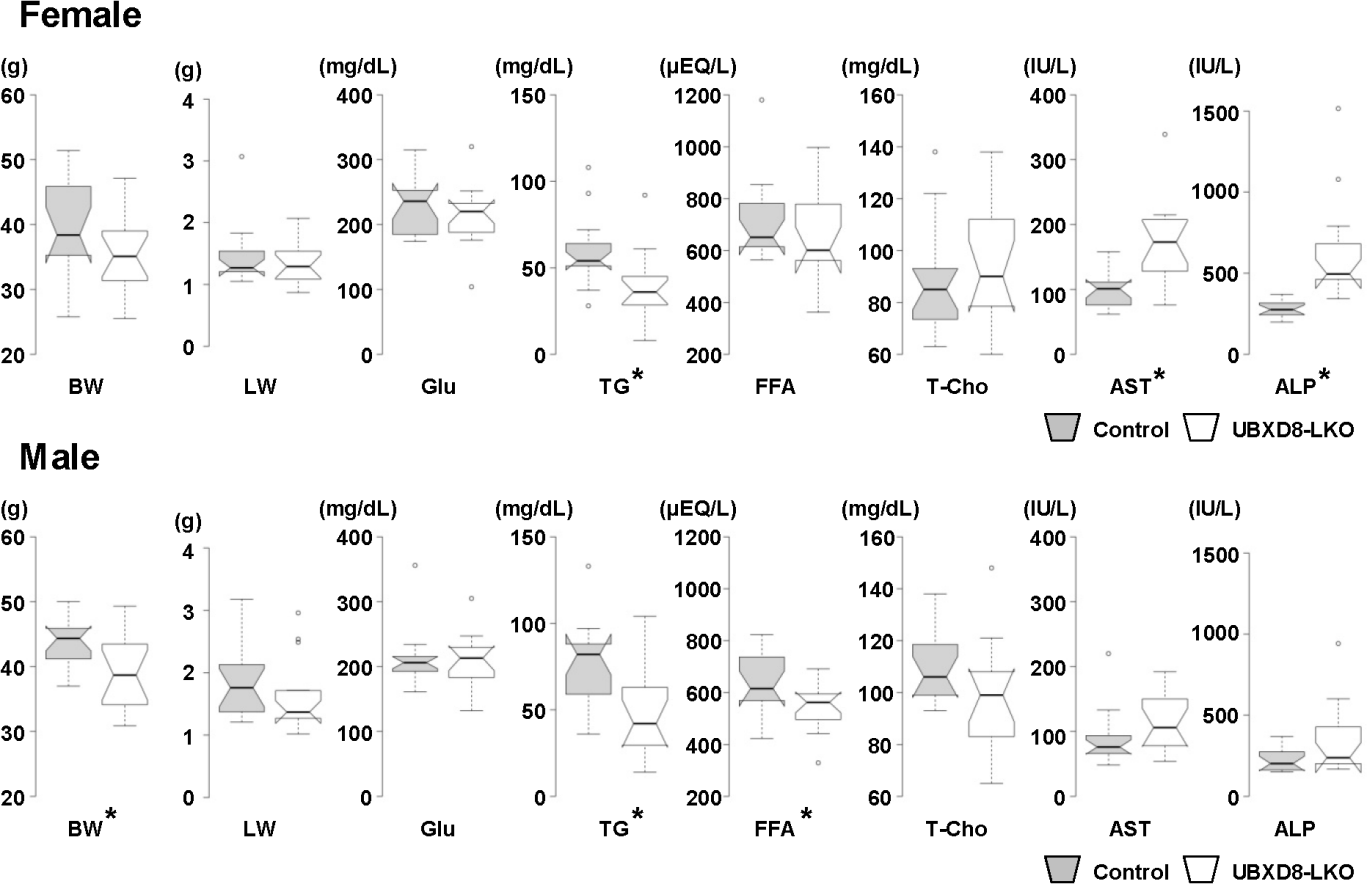
Body weight, liver weight, and blood chemistry results of mice fed a high-fat diet. Control and UBXD8-LKO mice (30 wk old) fed a high-fat diet for 26 wk. 15 females (A) and 15 males (B) were examined for each group. Indices marked with an asterisk (*) differed significantly between the two groups (P < 0.05; non-paired Student's t test). Box plots were prepared in the same manner as Fig 2. Numerical data (means ± SEM) and data on additional indices are shown in Table 2. BW: body weight; LW: liver weight; Glu: glucose; FFA: free fatty acid; T-Cho: total cholesterol.

When mice were fed a high-fat diet for 26 wk, from the time of weaning to the age of 30 wk, several significant differences were observed between UBXD8-LKO and control mice (Fig 3, Table 2). Under these conditions, as before, we examined 15 females and 15 males from each group, i.e., 60 mice in total. For many indices, statistically significant differences were seen only in females or males, but under these conditions a difference in the same direction also occurred in the other sex. Three major differences were observed. First, UBXD8-LKO mice had lower body weight than control mice (0.887-fold in females and 0.897-fold in males). Second, serum TG and free FA were lower in the UBXD8-LKO mice than in controls (TG: 0.665-fold in females and 0.632-fold times in males; free FA: 0.899-fold in females and 0.853-fold in males). Third, serum AST and ALP were higher in UBXD8-LKO mice than in control (AST, 1.74-fold in females and 1.30-fold in males; ALP, 2.23-fold in females and 1.52-fold in males).

**Table 2:** Body weight, liver weight, and blood chemistry results of mice fed a high-fat diet.

High-fat diet						
	Female			Male		
	Control	UBXD8-LKO	P value	Control	UBXD8-LKO	P value
**Parameter**						
**Body weight (g)**	**39.63±1.79**	**35.18±1.43**	**0.063**	**43.51±1.13**	**39.04±1.51**	0.025*
**Liver weight (g)**	**1.44±0.12**	**1.34±0.09**	**0.518**	**1.82±0.15**	**1.60±0.15**	**0.326**
**Liver/Body ratio (%)**	**3.61±0.19**	**3.79±0.16**	**0.512**	**4.17±0.30**	**4.03±0.24**	**0.729**
**Serum parameter**						
**Total protein (g/dL)**	**4.78±0.04**	**4.89±0.09**	**0.309**	**4.77±0.05**	**4.68±0.07**	**0.332**
**Albumin (g/dL)**	**3.25±0.04**	**3.28±0.05**	**0.621**	**3.04±0.04**	**2.95±0.05**	**0.24**
**Total bilirubin (mg/dL)**	**0.060±0.007**	**0.094±0.008**	0.005*	**0.050±0.002**	**0.057±0.003**	**0.147**
**Glucose (mg/dL)**	**230.53±11.66**	**213.53±12.12**	**0.321**	**211.40±11.47**	**208.87±10.42**	**0.871**
**Triglyceride (mg/dL)**	**59.67±5.14**	**39.67±4.93**	0.009*	**76.73±6.28**	**48.47±6.89**	0.005*
**Phospholipid (mg/dL)**	**162.07±8.69**	**183.60±9.34**	**0.103**	**203.87±6.33**	**187.00±8.45**	**0.122**
**Free fatty acid (μEQ/L)**	**724.20±41.04**	**651.27±43.04**	**0.23**	**639.87±32.13**	**545.67±24.26**	0.027*
**Total cholesterol (mg/dL)**	**88.93±5.58**	**94.07±5.81**	**0.529**	**110.13±3.78**	**97.73±5.45**	**0.072**
**Esterified cholesterol (mg/dL)**	**69.07±4.27**	**69.87±4.50**	**0.898**	**84.20±2.85**	**73.87±4.05**	0.046*
**Free cholesterol (mg/dL)**	**19.87±1.37**	**24.20±1.41**	0.037*	**25.93±1.02**	**23.87±1.46**	**0.257**
**Aspartate aminotransferase (IU/L)**	**99.67±7.16**	**173.53±16.36**	<0.0001*	**89.20±11.23**	**115.80±11.87**	**0.115**
**Alanine aminotransferase (IU/L)**	**44.40±8.86**	**85.87±9.27**	0.003*	**57.93±17.46**	**54.60±12.76**	**0.879**
**Lactate Dehydrogenase (IU/L)**	**570.73±84.71**	**486.87±40.20**	**0.379**	**478.93±49.36**	**513.93±54.61**	**0.638**
**Alkaline phosphatase (IU/L)**	**280.80±12.96**	**624.80±80.54**	<0.0001*	**226.87±19.98**	**344.07±55.82**	**0.058**

Comparison of control and UBXD8-LKO mice (30 wk old) fed a high-fat diet for 26 wk. For each group, 15 females and 15 males were examined. P values were obtained by non-paired Student's t test. Selected data sets are presented as box plots in Fig 3. n = 15. All data are presented as mean ± SE. P values are the results of non-paired Student's t test

(*: P<0.05).

**Fig 4:**
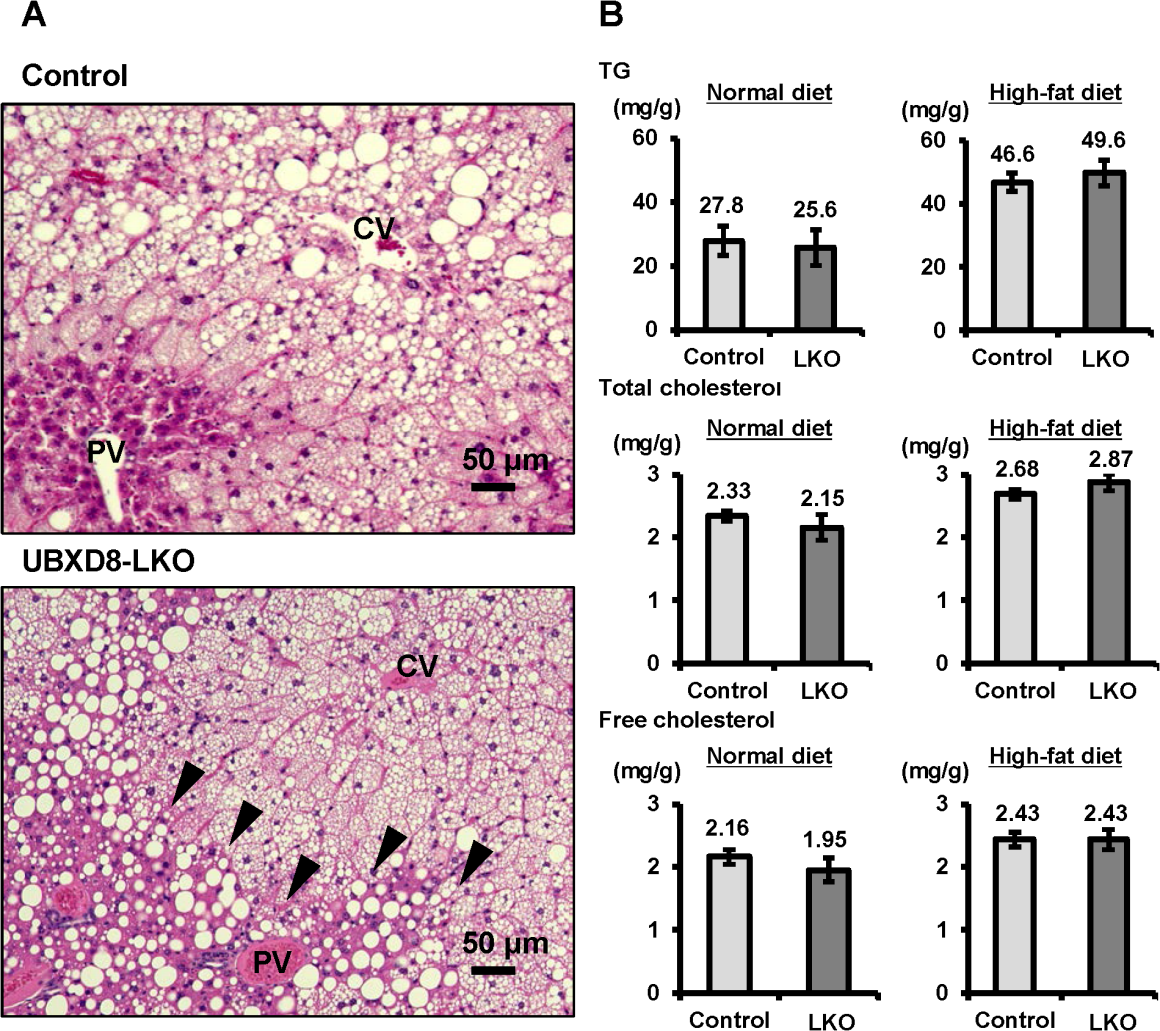
High-fat diet caused periportal steatosis in UBXD8-LKO mice. (A) Histology of liver sections obtained from control and UBXD8-LKO mice (30 wk old) fed a high-fat diet for 26 wk. The perivenular area (zone 3) of both groups showed steatosis of the predominantly microvesicular type, whereas the periportal area (zone 1) showed macrovesicular steatosis only in UBXD8-LKO mice. CV: central vein, PV: portal vein. (B) Analysis of lipids extracted from livers of control and UBXD8-LKO mice fed a high-fat diet for 26 wk. TG, total cholesterol, and free cholesterol did not exhibit significant differences between the two groups (n = 5; means ± SEM).

Liver histology also differed notably between UBXD8-LKO mice and controls. In both groups, microvesicular steatosis was prominent in the perivenular area (zone 3) and transition area (zone 2). However, macrovesicular steatosis in the periportal area (zone 1) was observed exclusively in UBXD8-LKO mice (Fig 4A). Electron microscopy confirmed that macrovesicular structures in the periportal hepatocytes of UBXD8-LKO mice were large LDs. These LDs were structurally similar to those in microvesicular steatotic cells, but were often surrounded by abundant glycogen granules (S2 Fig).

In UBXD8-LKO mice, periportal macrovesicular steatosis was seen more frequently in females than in males (Table 3), consistent with the finding that serum AST and ALP levels were much higher in females (Fig 3, Table 2). On the other hand, the amounts of TG, total cholesterol, and free cholesterol in liver did not differ significantly between UBXD8-LKO mice and controls, probably due to the presence of microvesicular steatosis in a wide area of the lobule in both groups (Fig 4B). Macrovesicular steatosis in UBXD8-LKO mice was not accompanied by inflammatory changes (Fig 4A) or fibrosis (data not shown). Insulin sensitivity was also unchanged between UBXD8-LKO and control mice (data not shown).

**Table 3:** Histological evaluation of liver at 30 wk of age.

	Control				UBXD8-LKO			
Feeding	Normal diet		High-fat diet		Normal diet		High-fat diet	
Sex	Female	Male	Female	Male	Female	Male	Female	Male
**Perivenular microvesicular steatosis**	**0/15**	**0/15**	**6/15**	**12/15**	**0/15**	**0/15**	**6/15**	**8/15**
**Periportal macrovesicular steatosis**	**0/15**	**0/15**	0/15*	**1/15**	**0/15**	**0/15**	9/15*	**4/15**

For both control and UBXD8-LKO mice, 15 females and 15 males were examined by microscopy of paraffin sections stained with H&E. Statistical significance of the data was analyzed by Fisher’s exact test.

*P<0.001 by Fisher's exact test.

### VLDL in UBXD8-LKO mice

**Fig 5:**
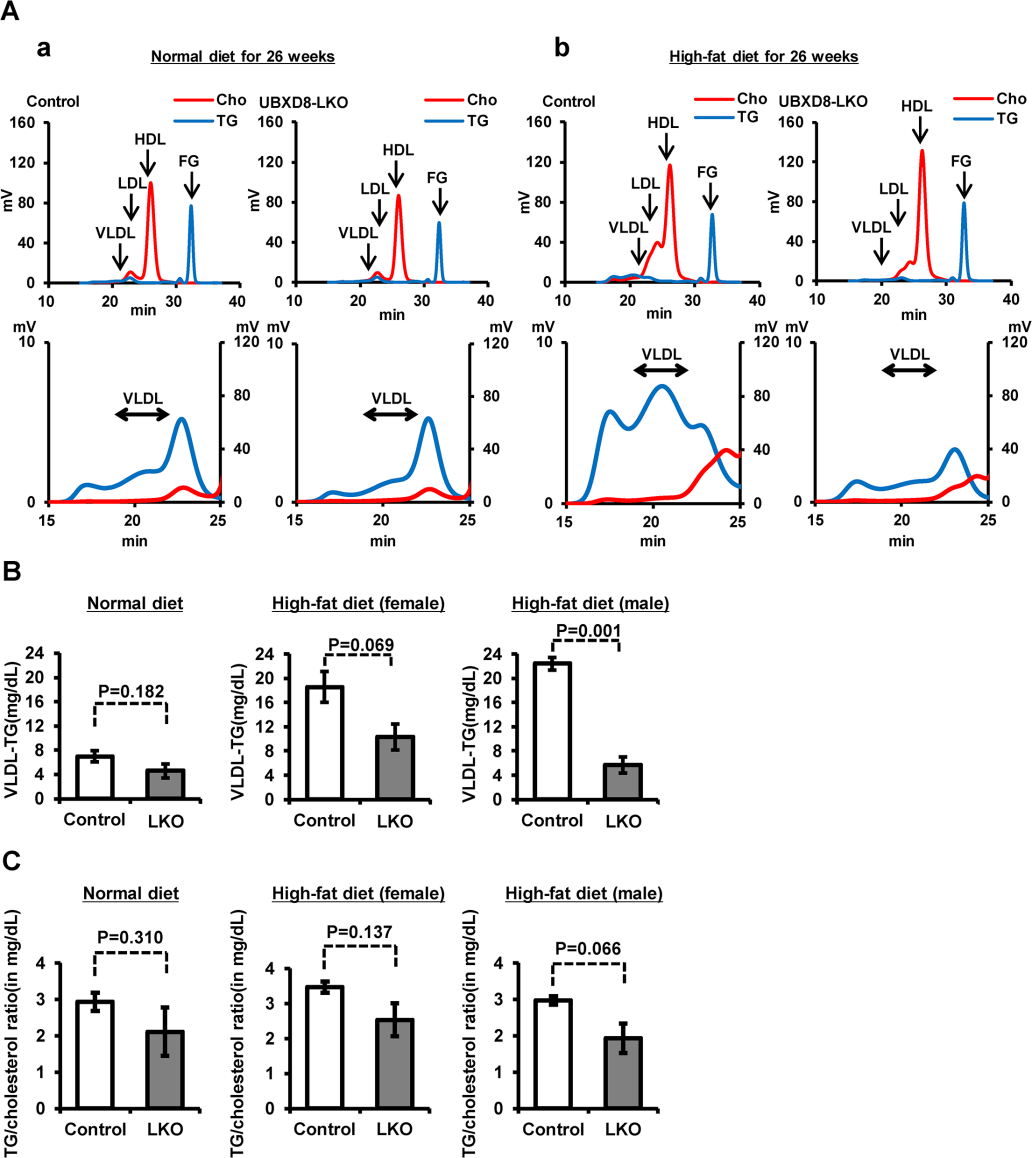
Analysis of serum lipoproteins. (A) Lipoprotein profile obtained by gel filtration–HPLC of mouse serum at 30 wk old. A representative result on TG (blue) and total cholesterol (red) is shown for sera of normal and UBXD8-LKO male mice fed a normal (A) or a high-fat diet (B). Sera from female mice gave similar results. Sera for the UBXD8-LKO group fed a high-fat diet were taken from mice showing periportal steatosis. Lower panels show a magnified view of the VLDL portion. FG: free glycerol. (B) VLDL-TG was lower in UBXD8-LKO mice than in normal mice, even when they were fed a normal diet. The difference between the control and UBXD8-LKO mouse became significantly larger when mice were fed a high-fat diet. P values were obtained by non-paired Student's t test (n = 3; means ± SEM). (C) The TG-to-cholesterol ratio in VLDL tended to be lower in the UBXD8-LKO than in the control mouse. P values were obtained by non-paired Student's t test (n = 3; means ± SEM).

In light of the finding that the serum TG level was significantly lower in UBXD8-LKO mice than controls under a high-fat diet, we analyzed the lipoprotein profile to measure TG in lipoprotein fractions (Fig 5A). TG in VLDL (VLDL-TG) was lower in UBXD8-LKO mice than in controls, and the difference became significantly larger when the animals were fed a high-fat diet (Fig 5B). The TG-to-cholesterol ratio in VLDL was also lower in UBXD8-LKO mice than in controls whether mice were fed a normal or a high-fat diet (Fig 5C). Considering that TG and cholesterol are largely present in the core and the outer phospholipid monolayer of the VLDL particle, respectively, these results suggested that lipidation of ApoB (i.e., loading of TG to nascent ApoB) is defective in the UBXD8-null hepatocytes.

**Fig 6:**
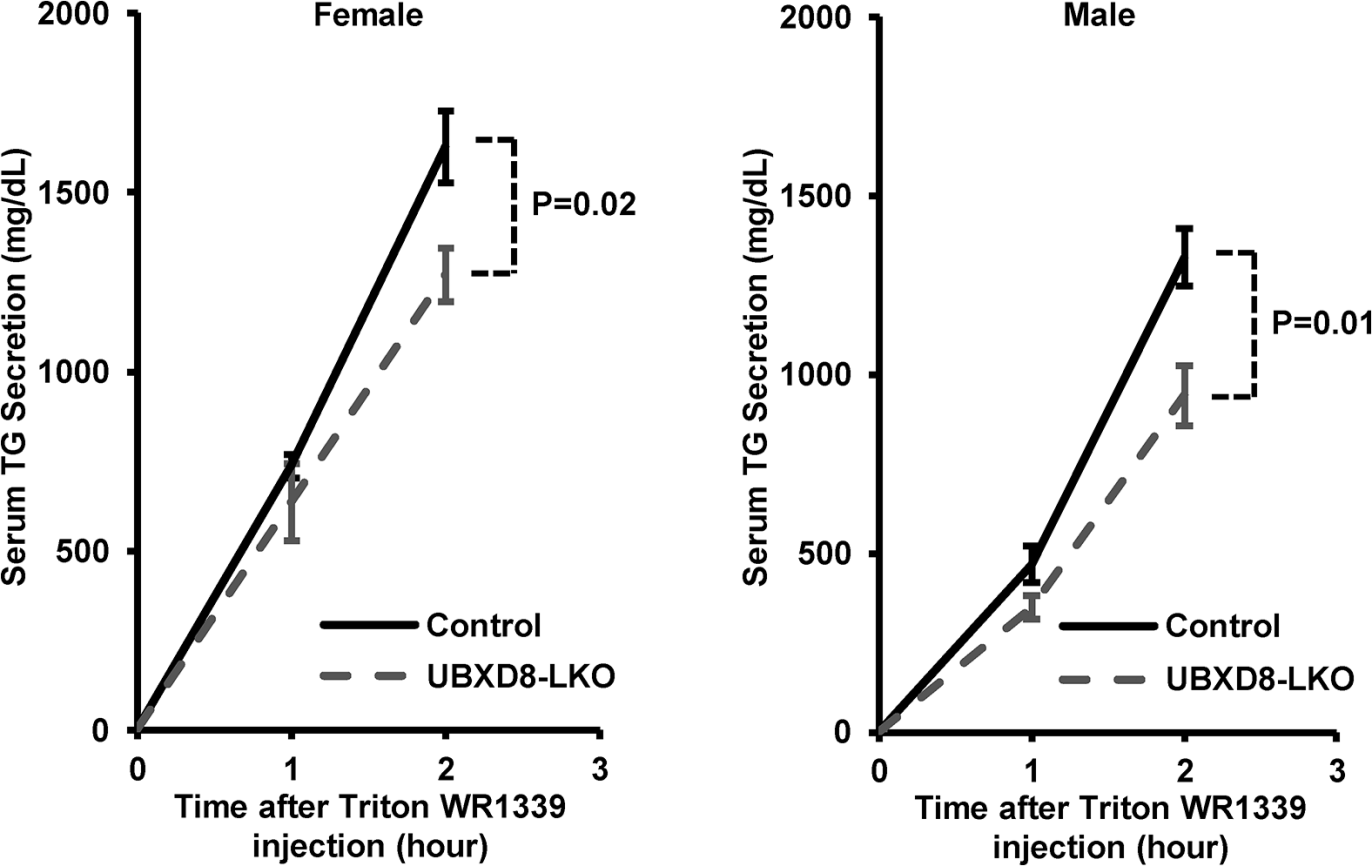
TG secretion was lower in UBXD8-LKO mice than in controls. TG secretion estimated by the Triton WR-1339 injection method. Control and UBXD8-LKO mice, both male and female, were fed a normal diet until 30–33 wk of age. Serum TG levels were measured before and 1 and 2 h after the Triton WR-1339 injection. P values were obtained by non-paired Student's t test (n = 4; means ± SEM).

The low TG level in UBXD8-LKO mice also suggested that the absence of UBXD8 suppressed secretion from hepatocytes. To address this possibility, we measured serum TG after injection of Triton WR-1339, which inhibits lipoprotein lipase and thereby suppresses lipoprotein degradation [21]. The result clearly showed that TG level after Triton WR-1339 injection was significantly lower in UBXD8-LKO mice than in controls, in both females and males (Fig 6), indicating that the absence of UBXD8 from hepatocytes led to a decrease in lipoprotein secretion.

We suspected that the lack of UBXD8 might have down-regulated VLDL secretion indirectly by influencing expression of other molecules. To investigate this possibility, we measured mRNAs related to VLDL synthesis. However, neither quantitative real time-PCR (S3 Fig) nor microarray analysis (S1 Table) revealed any significant difference between UBXD8-LKO mice and the controls. Thus, the observed decrease in serum VLDL-TG was caused by the loss of UBXD8 function in hepatocytes.

### UBXD8-deficient hepatocytes were decreased in ApoB secretion

**Fig 7:**
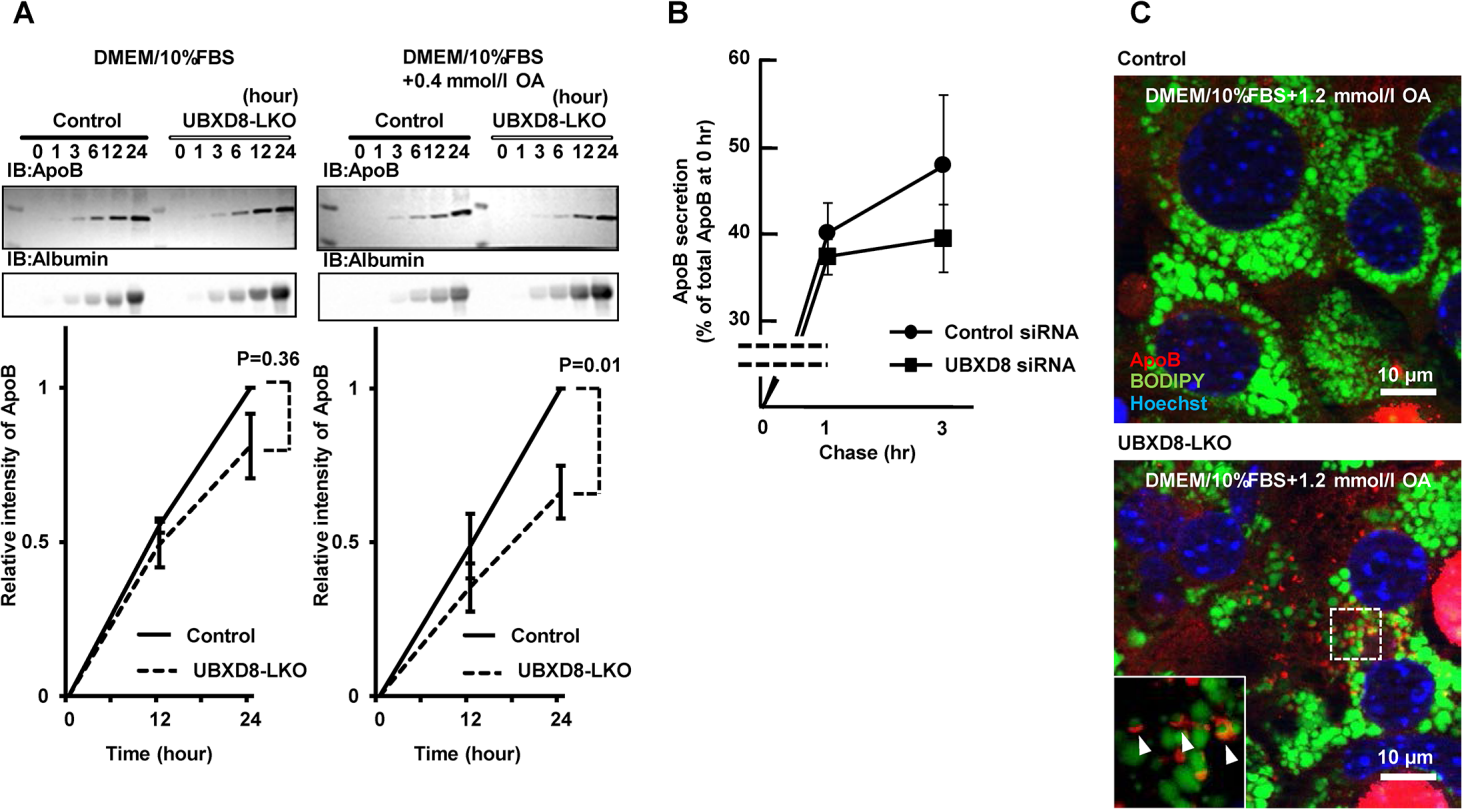
Comparison of cultured cells. (A) Cultured primary hepatocytes obtained from the UBXD8-LKO mice secreted lower levels of ApoB than cells obtained from the control mice. Hepatocytes obtained from female mice fed a normal diet until 12 wk of age were cultured, and ApoB and albumin secreted into the culture medium were examined by densitometry of Western-blot signals. The albumin signal was used for normalization. The amount of secreted ApoB was lower in hepatocytes obtained from UBXD8-LKO mice than in those obtained from control mice, whether the cells were cultured in the normal medium (DMEM + 10% FBS) (left) or in the medium supplemented with 0.4 mmol/l OA (right). P values were obtained by non-paired Student's t test (n = 3; means ± SEM). (B) HepG2 cells transfected with control or UBXD8 siRNA were pulse-labeled with ^*35*^S-methionine and treated with 0.4 mmol/l oleic acid. ApoB immunoprecipitated from the medium at 1 and 3 h was subjected to SDS-PAGE and quantitated by radiography. ApoB secreted in the medium is shown as the ratio relative to total cellular ApoB immediately after pulse-labeling. (C) Primary hepatocytes obtained from UBXD8-LKO mice showed ApoB-crescent structures. The ApoB-crescent was observed as a crescent-shaped ApoB labeling (red) adjacent to LDs (green). Nuclei were stained with Hoechst dye (blue). The ApoB-crescent was present only in cells derived from UBXD8-LKO mice, and not in those obtained from control mice. Cells were cultured in the presence of 1.2 mmol/l OA.

To more directly measure the effect of UBXD8 deficiency on VLDL secretion, we cultured hepatocytes isolated from livers of UBXD8-LKO and control mice, and compared the amount of ApoB secreted into the culture medium. Based on the *in vivo* result that the serum albumin level was equivalent between UBXD8-LKO and control mice (Tables 1 and 2), we used secreted albumin as the normalization standard. ApoB secretion from the UBXD8-deficient hepatocytes was lower than that from controls in standard culture medium, i.e., DMEM and 10% FBS (Fig 7A). The difference became more evident and significant when 0.4 mmol/l OA was added to the culture medium (Fig 7A). A similar decrease in ApoB secretion was observed in HepG2, a hepatocellular cell line that retains the ability to secrete ApoB [25], when UBXD8 was knocked down by siRNA transfection (Fig 7B). These results confirmed that the decrease in serum VLDL-TG in the UBXD8-LKO mouse was caused by downregulation of VLDL secretion from hepatocytes.

Finally, the ApoB-crescent, an LD–ER amalgamation structure that indicates aberrant accumulation of lipidated ApoB [14,15], was observed in primary hepatocytes obtained from UBXD8-LKO mice, but not in those from control mice (Fig 7C). Although the frequency of ApoB-crescents was low, this result indicated that UBXD8 in normal hepatocytes is also engaged in proteasomal degradation of lipidated ApoB, as shown previously in hepatocarcinoma cell lines [15,16].

## Discussion

In this study, we showed that hepatocyte-specific UBXD8-deficient mice fed a high-fat diet develop periportal macrovesicular steatosis accompanied by a decrease in VLDL secretion. The phenotype was distinct from that of the majority of mouse steatosis models generated by either dietary or genetic manipulation, in which blood TG levels are higher than or equivalent to those of controls [5,26].

Steatosis with abnormal VLDL secretion has been observed in several mouse models: mice fed a methionine- and choline-deficient (MCD) diet [27], liver-specific microsomal triglyceride transfer protein (MTP)-knockout mice [28], mice heterozygously expressing the ApoB 38.9 mutant [29], methionine adenosyltransferase 1A-knockout mice [30] and glycine N-methyltransferase-knockout mice [31]. In these models, VLDL secretion was reduced due to a defect in an early step of lipoprotein formation: the MCD diet as well as depletion of either methionine adenosyltransferase 1A impairs VLDL formation by suppressing phosphatidylcholine synthesis [32]; in the absence of glycine N-methyltransferase, a high level of S-adenosylmethionine disrupts VLDL assembly [31]; MTP depletion and the truncated ApoB mutant hinder co-translational processes by inhibiting lipidation [33] and perturbing TG packaging, respectively. Unlike other secretory proteins, ApoB secretion is regulated mainly by proteasomal degradation of poorly lipidated nascent polypeptide [11]. In both MTP-KO and ApoB 38.9 mutant mice, the reduction in VLDL was caused by activation of this mechanism.

**Fig 8:**
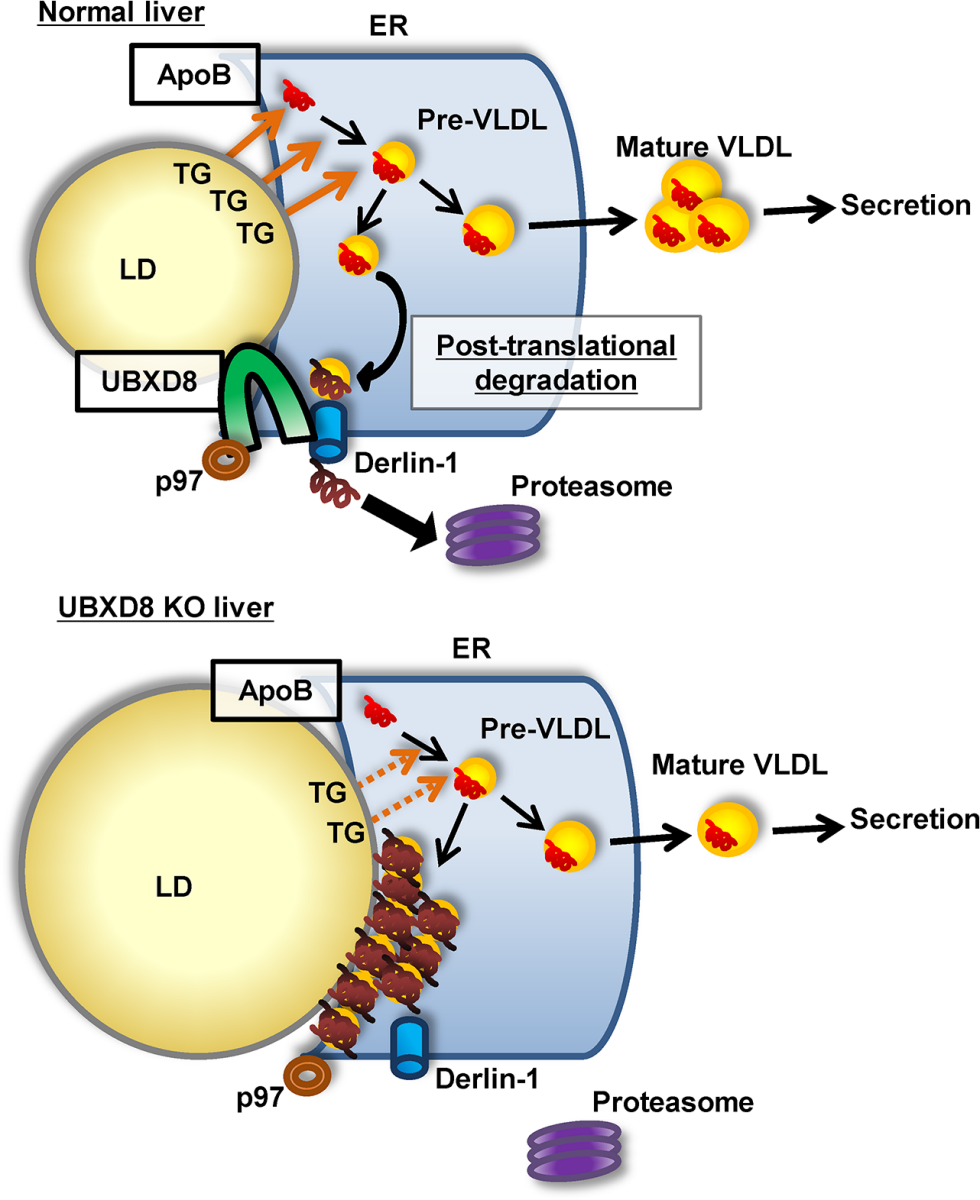
Diagram of ApoB lipidation and VLDL maturation in normal and UBXD8-deficient hepatocytes. In normal hepatocytes, lipidated ApoB destined for proteasomal degradation is translocated to the cytoplasmic side and subjected to ubiquitination by a UBXD8-dependent mechanism. In the absence of UBXD8, lipidated ApoB accumulates at the ER–LD interface and disturbs the normal ApoB lipidation process. This probably results in a decrease in VLDL-TG secretion in UBXD8-null hepatocytes.

What, then, was the cause of a similar abnormality, i.e., steatosis with decreased VLDL secretion, in UBXD8-LKO mice? Our previous study showed that UBXD8 plays a critical role in degrading ApoB after lipidation [16], which is distinct from the co-translational mechanism that deals with poorly lipidated ApoB at the translocon [11,13]. We also showed that knockdown of UBXD8 induces accumulation of lipidated ApoB on the LD surface facing the ER lumen [16]. Furthermore, we found that serum VLDL in the UBXD8-LKO mouse showed a lower TG-to-cholesterol ratio than did that in the control mouse (Fig 5C), suggesting a defect in the ApoB lipidation process. Based on these results, we speculate that the abnormalities that we observed in the UBXD8-LKO mouse were caused as follows. When the UBXD8-dependent ApoB degradation mechanism is compromised, lipidated ApoB accumulates at the interface between LDs and the ER [15,16]. This aberrant ApoB accumulation is likely to disturb the normal lipidation process, which involves utilization of lipids in LDs at the LD–ER interface [8], eventually leading to a decrease in VLDL secretion (Fig 8). On the other hand, an *in vitro* study indicated that UBXD8 functions as a sensor for unsaturated FA, thereby regulating TG synthesis [17]. Lack of this functionality may contribute to the development of steatosis, but it is not likely to cause the decrease of serum TG, VLDL, and ApoB secretion that was observed in the UBXD8-LKO mouse.

When the UBXD8-LKO mice were fed a normal diet, they did not exhibit any difference relative to controls. A high-fat diet induced macrovesicular steatosis in the periportal region of the UBXD-deficient liver, but even in this condition, neither inflammation nor fibrosis was observed. The mild phenotype of the UBXD8-LKO mouse suggests that the UBXD8-dependent mechanism plays a relatively minor role in intracellular ApoB degradation. When the UBXD8-dependent mechanism is not working normally, it is likely that other post-translational ApoB degradation mechanisms, which may be collectively called "post-ER presecretory proteolysis" [12], are up-regulated, so that the abnormality became apparent only upon exposure to an additional stress (e.g., a high-fat diet). Nevertheless, the results of this study show for the first time that a defect in a degradation mechanism of ApoB after lipidation could lead to steatosis.

Notably, UBXD8-LKO mice fed a high-fat diet developed steatosis in the periportal zone, whereas control mice did not. Periportal steatosis was previously observed in ATGL-knockout mice [34] and CGI58-knockout mice [35]. ATGL and its activator, CGI58, are engaged in hydrolyzing TG to generate FAs [36]; consequently, preferential LD accumulation in periportal hepatocytes in mice lacking either ATGL or CGI58 might suggest zonation of the liver lobule with respect to TG hydrolysis activity [37,38]. There is no consensus view on zonation in the normal liver in regard to VLDL secretion [38,39]; however, upon exposure to endotoxin, VLDL secretion is up-regulated, primarily in periportal hepatocytes [40]. This observation suggests that periportal hepatocytes have a greater potential to produce VLDL than hepatocytes in other zones. We speculate that the increase in VLDL secretion in animals receiving a high-fat diet may also be carried out preferentially by periportal hepatocytes. When VLDL secretion increases, the levels of aberrant ApoB that needs to be degraded by post-translational mechanisms should also increase. In this study, periportal steatosis in the UBXD8-LKO mouse probably became evident due to this additional requirement imposed by a high-fat diet.

In human, periportal steatosis is observed only in a limited number of diseases, such as AIDS, malnutrition, and kwashiorkor [41]. It is rare in adult non-alcoholic fatty liver disease (NAFLD), but it occurs with a higher frequency in pediatric NAFLD [42]. It is not clear why steatosis in these diseases occurs in the periportal zone, but the results reported here, as well as the results of ATGL-knockout and CGI58-knockout mouse studies, might provide clues about pathogenesis.

Another notable observation in this study was that female UBXD8-LKO mice exhibited abnormalities in more indices than male mice. A similar gender difference has been observed in a few other non-alcoholic steatohepatitic (NASH) mouse models, but in most others, especially those induced by a high-fat diet, males exhibit more severe phenotype than females [43]. On the other hand, although some studies have shown that NASH in human patients above 50 years old is more prevalent in women than in men, other studies have reported that the male–female distribution of NASH varies by ethnicity [44]. Thus, many uncertainties remain regarding whether and how gender affects the incidence of steatosis and related liver diseases. These observations emphasize the importance of examining mice of both sexes, especially when studying genetically engineered mouse models for the first time.

The amino-acid sequences of human and mouse UBXD8 are almost identical (97.75% identity, 99.32% similarity), but steatosis due to UBXD8 dysfunction has not been reported in humans. We suspect that this is because UBXD8 is expressed in many organs, so that mutations that severely compromise its functionality may be fatal. Indeed, we found that systemic depletion of UBXD8 in mouse is lethal at an early stage of embryonic development (Suzuki and Fujimoto, unpublished observation). On the other hand, mutations with relatively mild effects on UBXD8 function may not cause apparent phenotypes. However, individuals with such UBXD8 mutations may be predisposed to diseases, which manifest under unfavorable conditions. Therefore, we believe that UBXD8 mutations in human samples warrant further studies.

## Supporting Information

S1 Fig Histology of liver sections obtained from control and UBXD8-LKO mice (30 wk old) fed a normal diet for 26 weeks. No pathological changes were observed in either group. CV: central vein, PV: portal vein.
(DOCX)

S2 Fig Electron microscopy of hepatocytes *in vivo*. (A) Hepatocytes in the perivenular zone of control mice. Cells had many LDs of less than 2 μm in diameter. (B) Hepatocytes in the periportal zone of UBXD8-LKO mice. Large LDs, often more than 5 zm in diameter, were observed in close association with clusters of glycogen granules.
(DOCX)

S3 Fig Quantitative real-time PCR analysis. Liver samples were examined from 30-wk-old female mice fed a normal or a high-fat diet. The level of gene expression was normalized using Hprt as the standard (means ± SEM). No significant difference was observed in any mRNA.
(DOCX)

S1 Table DNA microarray analysis. Liver samples were taken from control and UBXD8-LKO female mice of 30 wk old, which were fed a normal diet or a high-fat diet, and exhibited the typical histological feature as shown in Fig 4A and S1 Fig. One mouse was used for each experimental group.
(XLS)
