# Correction: Hepatocyte-Specific Depletion of UBXD8 Induces Periportal Steatosis in Mice Fed a High-Fat Diet

**DOI:** 10.1371/journal.pone.0148004

**Published:** 2016-01-25

**Authors:** Norihiro Imai, Michitaka Suzuki, Kazuhiko Hayashi, Masatoshi Ishigami, Yoshiki Hirooka, Takaya Abe, Go Shioi, Hidemi Goto, Toyoshi Fujimoto

There is an error in the second sentence in the PCR section of Materials and Methods. The correct sentence is: Primers were as follows: for loxP, forward primer: 5’-CCAACCTCTGTGGGTCCTC-3’; reverse primer: 5’-GCGATTGGGGATCTGAGTTA-3’; for albumin-Cre, forward primer: 5’-TTTGCCTGCATTACCGGTCGATGCAAC -3’; reverse primer: 5’-TGCCCCTGTTTCACTATCCAGGTTACGGA-3’.

## References

[1] Imai N, Suzuki M, Hayashi K, Ishigami M, Hirooka Y, Abe T, et al (2015) Hepatocyte-Specific Depletion of UBXD8 Induces Periportal Steatosis in Mice Fed a High-Fat Diet. PLoS ONE 10(5): e0127114 doi: 10.1371/journal.pone.0127114 25970332 10.1371/journal.pone.0127114 PMC4430229

