# Supplementary material for: Hepatocyte-Specific Depletion of UBXD8 Induces Periportal Steatosis in Mice Fed a High-Fat Diet
Source: PLoS One. 2015 May 13;10(5):e0127114. doi: 10.1371/journal.pone.0127114 (PMC4430229; doi:10.1371/journal.pone.0127114)
Supplement: S1 Fig — No pathological changes were observed in either group. CV: central vein, PV: portal vein. (DOCX) [file pone.0127114.s001.docx]

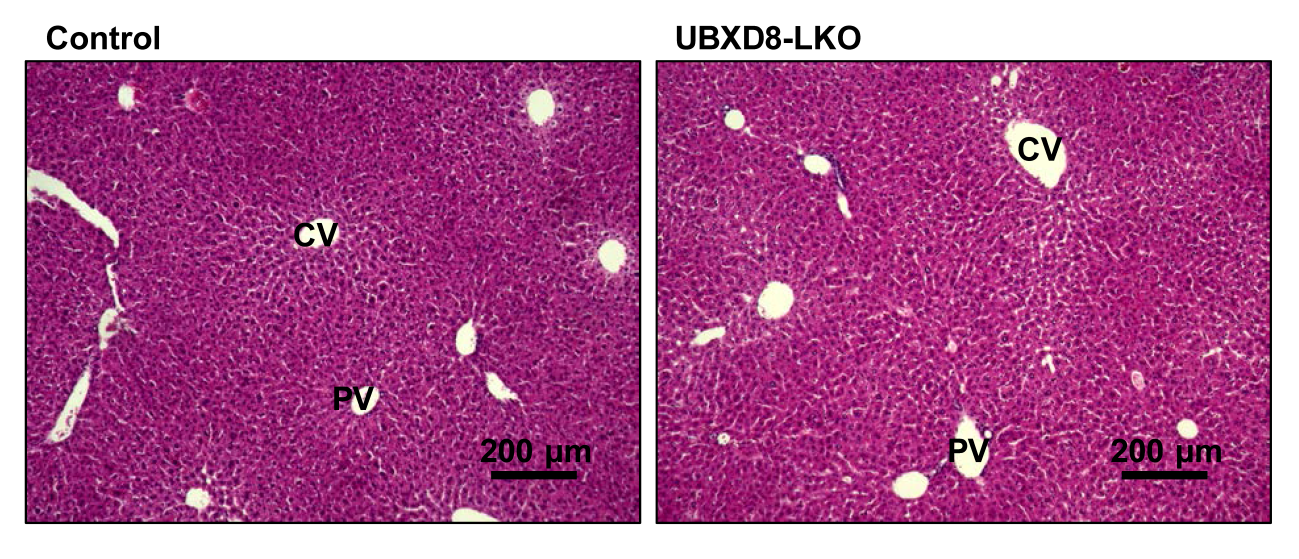


**S1 Fig. Histology of liver sections obtained from control and UBXD8-LKO mice (30 weeks old) fed a normal diet for 26 weeks.**

No pathological changes were observed in either group. CV: central vein, PV: portal vein.
