# Supplementary material for: Hepatocyte-Specific Depletion of UBXD8 Induces Periportal Steatosis in Mice Fed a High-Fat Diet
Source: PLoS One. 2015 May 13;10(5):e0127114. doi: 10.1371/journal.pone.0127114 (PMC4430229; doi:10.1371/journal.pone.0127114)
Supplement: S2 Fig — (A) Hepatocytes in the perivenular zone of control mice. Cells had many LDs of less than 2 μm in diameter. (B) Hepatocytes in the periportal zone of UBXD8-LKO mice. Large LDs, often more than 5 zm in diameter, were observed in close association with clusters of glycogen granules. (DOCX) [file pone.0127114.s002.docx]

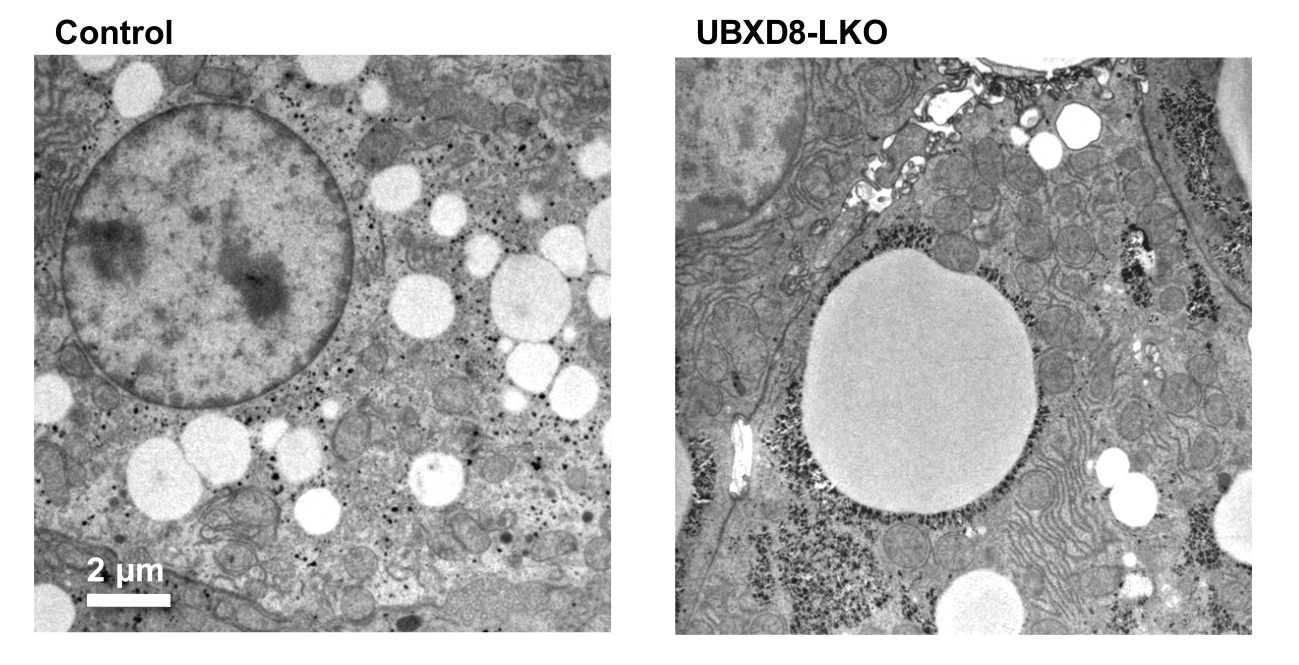


**S2 Fig. Electron microscopy of hepatocytes *in vivo*.**

(A) Hepatocytes in the perivenular zone of control mice. Each cell had many LDs of less than 2 μm in diameter. (B) Hepatocytes in the periportal zone of UBXD8-LKO mice. Large LDs, often more than 5 μm in diameter, were observed in close association with clusters of glycogen granules.
