# Supplementary material for: Hepatocyte-Specific Depletion of UBXD8 Induces Periportal Steatosis in Mice Fed a High-Fat Diet
Source: PLoS One. 2015 May 13;10(5):e0127114. doi: 10.1371/journal.pone.0127114 (PMC4430229; doi:10.1371/journal.pone.0127114)
Supplement: S3 Fig — Liver samples were examined from 30-wk-old female mice fed a normal or a high-fat diet. The level of gene expression was normalized using Hprt as the standard (means ± SEM). No significant difference was observed in any mRNA. (DOCX) [file pone.0127114.s003.docx]

**S3 Fig. Quantitative real-time PCR analysis.**

Liver samples were examined from 30-week-old female mice fed a normal or high-fat diet. The level of gene expression was normalized using *Hprt* as the standard (means ± SEM). No significant difference was observed in any mRNA.
